# Nonsynonymous Mutations in Linker-2 of the Pdr5 Multidrug Transporter Identify a New RNA Stability Element

**DOI:** 10.1534/g3.119.400863

**Published:** 2019-11-22

**Authors:** Hadiar Rahman, Andrew Rudrow, Joshua Carneglia, Sister Stephen Patrick Joly, Dante Nicotera, Michael Naldrett, John Choy, Suresh V. Ambudkar, John Golin

**Affiliations:** * Department of Biology, Catholic University of America, Washington, DC 20064,; † Center for Biotechnology, University of Nebraska, Lincoln 68588, and; ‡ Laboratory of Cell Biology, Center for Cancer Research, NCI, NIH, Bethesda, MD 20892

**Keywords:** ABC transporter, nonsynonymous mutation, mRNA, Pdr5, drug resistance

## Abstract

Analysis of synonymous mutations established that although the primary amino acid sequence remains unchanged, alterations in transcription and translation can result in significant phenotypic consequences. We report the novel observation that a series of nonsynonymous mutations in an unconserved stretch of amino acids found in the yeast multidrug efflux pump Pdr5 increases expression, thus enhancing multidrug resistance. Cycloheximide chase experiments ruled out the possibility that the increased steady-state level of Pdr5 was caused by increased protein stability. Quantitative-RT PCR experiments demonstrated that the mutants had levels of *PDR5* transcript that were two to three times as high as in the isogenic wild-type strain. Further experiments employing metabolic labeling of mRNA with 4-thiouracil followed by uracil chasing showed that the half-life of *PDR5* transcripts was specifically increased in these mutants. Our data demonstrate that the nucleotides encoding unconserved amino acids may be used to regulate expression and suggest that Pdr5 has a newly discovered RNA stability element within its coding region.

Broad-spectrum resistance to antibiotics in pathogens and to chemotherapeutic agents in tumor cells remains a major clinical problem. Multiple mechanisms are responsible for this, with genetic alterations in ATP-binding cassette (ABC) multidrug transporter**s** playing a major role. Often, multidrug resistance results from overexpression of these proteins in fungal pathogens and cancers (Pfaller 2002; Gottesman, Fojo, and Bates 2002; Prasad *et al.* 2014; Kathawala *et al.* 2015).

The yeast multidrug transporter Pdr5 has been the object of genetic and biochemical analyses since its discovery in 1990 (see Golin and Ambudkar, 2015 for review). It is the founding member of a large, clinically relevant subfamily of fungal efflux pumps. Mutations leading to *PDR5* overexpression create hyper-resistance to many structurally and mechanistically distinct xenobiotic compounds. Significantly, additional mutations can further increase drug resistance 2-4 times without changing the level of expression (Downes *et al.* 2013; Arya *et al.* 2019). Phenotypically similar mutants also exist in Cdr1, a Pdr5 homolog with 53% amino acid identity (Kolaczkowski *et al.* 2013; Tanabe *et al.* 2019).

Bioinformatic analysis of Pdr5 indicates that it has very long and relatively unconserved linker regions that connect portions of the transmembrane domains (TMDs) with the nucleotide-binding domains (Rutledge *et al.* 2011). These parts of the Pdr5 transporter have not been studied to date. In the structurally similar ABCG5/ABCG8 asymmetric mammalian lipid transporter, an R263Q mutation in the long linker connecting transmembrane helix 1 (TMH-1) of ABCG8 to the nucleotide-binding domain (NBD) has a loss-of-function phenotype resulting in sitosterolemia (Heimer *et al.* 2002). Linker 2 of Pdr5, which extends from TMH-6 to the canonical portion of NBD2, caught our attention. This linker contains a series of six serine residues that appeared as phosphopeptides in four mass spectrometry studies of yeast phosphorylation sites (Chi *et al.* 2007; Li *et al.* 2007; Albuquerque *et al.* 2008; Holt *et al.* 2009). A relatively early study of Pdr5 indicated that phosphorylation of the transporter is mediated by overlapping casein kinase-1 isoforms Yck1 and Yck2. The double mutant is a temperature-sensitive lethal that exhibited reduced localization of Pdr5 to the plasma membrane (Decottignes *et al.* 1999). Several residues in linker-2 are targets of these kinases. The role of phosphorylation in regulating ABC protein activity varies depending on the transporter or channel. In the case of the cystic fibrosis transmembrane conductance regulator, phosphorylation of its regulatory region is central to channel function (Gadsby and Nairn 1999; Mense *et al.*, 2006). This also seems to be a feature of other members of the ABCB1 family (Aryal, Laurent, and Geisler 2015). In contrast, phosphorylation of P-glycoprotein (P-gp) does not create an obvious phenotypic change in function (Germann *et al.* 1996).

To further explore the role of phosphorylation of Pdr5, we constructed single-alanine substitutions in each of the six residues found in linker-2. The resulting mutants exhibited strong multidrug hyper-resistance and enhanced whole-cell rhodamine 6G (R6G) drug transport. Western blotting of proteins from mutant plasma membrane (PM) vesicles clearly showed higher levels of Pdr5 than in the wild-type (WT) control.

It soon became apparent, however, that a lack of phosphorylation is not responsible for the hyper-resistant phenotype of the mutants. Mass spectrometry revealed that Ser-837 was only rarely phosphorylated. Furthermore, phosphomimic mutant S837D had a hyper-resistant phenotype that was similar to the alanine substitution. Additional experiments suggested that neither enhanced trafficking nor increased half-life of these mutant proteins could reasonably explain the phenotype. In light of these results, we considered the possibility that these nonsynonymous mutations were responsible for altering transcription or translation of the *PDR5* gene.

It is well known that synonymous mutations can alter phenotypes through a variety of mechanisms affecting transcription or translation (Duan *et al.* 2003; Chamary *et al.* 2005; Pagani, Raponi, and Baralle 2005; Kimchi-Sarfaty *et al.* 2007; Hunt *et al.* 2014; Gotea *et al.* 2015; Rauscher and Ignatova 2018). Synonymous mutations can result in either loss- or gain-of-function mutations. Synonymous mutations also alter the mammalian drug transporter P-gp (Kimchi-Sarfaty *et al.* 2007; Fung *et al.* 2014). Synonymous gain-of-function mutations also drive tumor production (Gotea *et al.* 2015).

In this study, we demonstrate that nonsynonymous mutations in the nucleotides coding for a large stretch of unconserved residues in the Pdr5 linker 2 region create drug hyper-resistance by increasing the half-life of the *PDR5* transcript. This strongly suggests that *PDR5* has a newly discovered RNA stability element in its coding region that limits the expression of this transporter.

## Materials and Methods

### Plasmids and Yeast strains

Table 1 shows the plasmids and Table 2 the yeast strains used in this study. Except for JG2001, all of the *Saccharomyces cerevisiae* strains were derived from R-1, which lacks all PM ABC transporters and contains a *PDR1-3* mutation that causes overexpression of *PDR5* when this gene is inserted at its chromosomal location. Thus, virtually all drug resistance to the particular compounds that we tested was mediated by the Pdr5 efflux pump. This strain offers other advantages for genetic and biochemical analyses, which are described in detail elsewhere (Sauna *et al.* 2008; Ananthaswamy *et al.* 2010). We cultured the strains at 30°. We used the pSS607-integrating plasmid for site-directed mutagenesis, as previously described (Golin *et al.* 2007). This plasmid has a WT *PDR5* gene under the transcriptional control of its own upstream region, as well as a *URA3*-selectable marker. Unless otherwise noted, we cultured cells with YPD medium at 30°.

**Table 1:** Plasmids used in this study

Plasmid designation	Pdr5 mutation	Reference
**pSS607**	Wild type	Golin *et al.* 2007
**pHR-1**	S837A	This study
**pHR-2**	S837D	This study
**pHR-3**	S840A	This study
**pHR-4**	S841A	This study
**pHR-5**	S849A	This study
**pHR-6**	S850A	This study
**pHR-7**	S854A	This study
**pHR-8**	S837A, S854A	This study
**pHR-9**	S837A, S850A	This study

**Table 2:** Yeast strains used in this study

Strain designation	Genotype	Reference
**R-1***^a^*	*MATα his1*, *ura3*, *PDR1-3*, *pdr5*::*KanMX4*, *snq2*, *yor1*, *pdr3*, *pdr10*, *ycf1*	Sauna *et al.* 2008 Derived from AD1-7
**JG2001**	*MATα his1*, *ura3*, *PDR1-3*, *PDR5*, *snq2*, *yor1*, *pdr3*, *pdr10*, *ycf1* The Δ*pdr5* gene in AD1-7 replaced by *PDR5*	Golin *et al.* 2007
**JG2015**	Isogenic to R-1 but has the addition of pSS607containing wild-type *PDR5* and *URA3* genes	Sauna *et al.* 2008
JG2015*^b^	Retransformation of R-1 with pSS607, but selection on 5-foa medium of a *ura3*, *PDR5* strain (no longer contains *KanMX4*).	This study
**JG2063**	Isogenic to R-1, but containing a G312A mutation	Furman *et al.* 2013
**JG2133**	Isogenic to R-1, but containing an A666G mutation	Arya *et al.* 2019
**JG2175**	Isogenic to R-1, but containing an S837A mutation	This study
**JG2176**	Isogenic to R-1, but containing an S837D mutation	This study
**JG2177**	Isogenic to R-1, but containing an S840A mutation	This study
**JG2178**	Isogenic to R-1, but containing an S841A mutation	This study
**JG2179**	Isogenic to R-1, but containing an S849A mutation	This study
**JG2180**	Isogenic to R-1, but containing an S850A mutation	This study
**JG2181**	Isogenic to R-1, but containing an S854A mutation	This study
**JG2182**	Isogenic to R-1, but containing S837A, S854A mutation	This study
**JG2183**	Isogenic to R-1, but containing S837A, S850A mutation	This study

a All site-directed mutations were made in pSS607 (Golin *et al.* 2007) and introduced into the R-1 strain by transformation. The desired *ura3* derivatives were selected on 5-foa medium and verified as described previously (Ananthaswamy *et al.* 2010).

b JG2015* is a recreation of JG2015 created specifically for this study.

### Chemicals and media

We purchased most of our chemicals from Sigma Aldrich. We obtained 5-fluoroorotic acid and G-418 from Research Products International and climbazole, cerulenin, cyproconazole, tebuconazole, and imazalil sulfate from LKT laboratories. We purchased tripropyltin chloride from Alfa Aesar. All chemicals were dissolved in DMSO except for 5-fluoroorotic acid and G-418, which were dissolved in sterilized YPD medium, and cycloheximide, which was dissolved in sterile water.

### Measurement of the relative resistance of strains to Pdr5 transport substrates

We placed 2 ml of YPD broth into sterile glass tubes. We introduced the desired concentration of drug into the tubes and added 0.5 x10^5^ cells (typically 2–5 µl). After culturing for 48 h, we measured absorbance at 600 nm (A_600_). We used isogenic WT and *∆pdr5* strains for comparing the susceptibility of the mutants to the drugs. For each strain, an untreated culture served as a control. We performed statistical analyses of the curves using Prism GraphPad Software.

### Site-directed mutagenesis

We introduced the nucleotide substitutions into pSS607 with a Quikchange Lightning site-directed mutagenesis kit (Life Technologies). We designed mutant primers with a genomics program provided by Agilent Technologies. The mutant plasmids were introduced into XL-Gold *E. coli* by transformation, as described in the Quikchange instruction manual. We extracted plasmid DNA from the transformants with an IBI miniprep kit (MIDSCI) and had it sequenced commercially to confirm the presence of the mutation in the plasmid (SeqWright). We introduced the mutant plasmid DNA into R-1 with a Sigma Aldrich yeast transformation kit. Genetic testing described elsewhere (Ananthaswamy *et al.* 2010) confirmed that the construct was correctly inserted.

### Preparation of purified PM vesicles

We adopted the procedure of Kolaczkowski *et al.* (1996) with minor modifications (Ernst *et al.* 2008). This protocol significantly reduces the amount of contaminating mitochondrial membrane and gives consistently higher ATPase activity.

### Preparation of whole-cell lysates

We prepared whole-cell lysates as previously described (Rahman *et al.* 2018).

### Gel electrophoresis of PM vesicle proteins

We solubilized samples containing 5 μg PM vesicle protein in SDS–PAGE for 30 min at 37°. We separated the proteins on NuPAGE 7% tris acetate gels (125–150 V for ∼70 min; Life Technologies).

Gels were stained with SimplyBlue (Coomaissie blue R-250) Safe Stain.

### Western blots of Pdr5 in PM vesicles and whole-cell extracts

We conducted Western blotting with 5 μg PM vesicle protein separated by gel electrophoresis. We performed the transfer from the gel to the nitrocellulose membrane (400 mAmp, 60 min) with an X Cell II minicell apparatus (Invitrogen). We used a custom-made Pdr5 rabbit antibody made against residues 813-831 in linker-2 by Thermo Fisher. Membranes were blocked for 40 min in PBS buffer with 1% tween (PBST) and 5% nonfat milk (NPBST) and then incubated at 4° overnight with gentle rocking in Pdr5 rabbit polyclonal antibodies (Thermofisher Scientific) and Pma1 mouse monoclonal antibody (Abcam) diluted 1:5000 and 1:10,000, respectively. Membranes were washed 3 times in PBST for 15 min before incubating at room temperature with gentle rocking for 2 hr in a 1:10,000 dilution of horseradish peroxidase (HRP) conjugated anti-rabbit IgG for Pdr5 (Sigma) and a 1:3000 dilution of anti-mouse specific for Pma1 (Abcam) in NPBST. We developed the blots with a Novex ECL horseradish peroxidase chemiluminescent substrate reagent kit (Invitrogen). The membrane was incubated in developer for 1 min and then scanned with ChemiDoc Touch (Bio Rad). We used Image J software (NIH) to analyze the bands.

To perform Western blots on whole-cell extracts, we solubilized proteins and performed gel electrophoresis with 10 μg of solubilized protein as described above. We ran the samples on 7% tris-acetate gels. We performed Western blots as described above. For a loading control, we used GAPDH primary antibody diluted 1:5000 and incubated at 4° overnight. The GAPDH secondary antibody was diluted 1:7000 and the filter incubated at room temperature for 1.5 h.

### Cycloheximide chase experiments

Pellets containing 1.3 × 10^9^ exponentially growing yeast cells were resuspended in 300 ml of fresh YPD medium, and cycloheximide was added to a final concentration of 125 μM at 2-min intervals. The cells were incubated at 30°. Fifty-ml samples were removed from each flask at intervals of 0, 30, 60, 120, 180, 240, and 300 min. The samples were immediately placed on ice. We performed the whole- cell lysis as described by Rahman *et al.* (2018) but did not add PMSF to the cell cultures prior to lysis. Gel electrophoresis and Western blotting were performed as described above.

### Assay of ATPase activity

We measured Pdr5-specific ATPase activity for 8 min at 35° with 2 µg purified PM vesicle protein in Tris-glycine (pH 9.5) buffer as previously described (Arya *et al.* 2019). The non-Pdr5 activity observed in the ΔPdr5 negative control strain was subtracted as background before calculating activity. We verified all ATPase results by carrying out assays with at least two independent PM vesicle preparations per strain.

### R6G transport in whole cells

We measured R6G transport against a 10-µM concentration gradient. We placed 3 × 10^6^ cells in 500 µl of 0.02 M Hepes, 1 mM glucose (pH 7.0), and 10 µM R6G and incubated them at 30° for 90 min. The cells were pelleted and washed with 1 ml of cold 0.02M Hepes buffer (pH 7.0) minus glucose. The pellets were resuspended in 500 µl of the same buffer and analyzed with a FACSort set to an excitation wavelength of 529 nm and an emission wavelength of 553 nm. We analyzed the data with a CellQuest program. We expressed retained fluorescence in arbitrary units (a.u.).

### Spectrometry of Pdr5 phosphopeptides

Pdr5 pm vesicle proteins were solubilized in 5X SDS PAGE buffer. We loaded 40 µg of protein into each well and separated the proteins by electrophoresis. The in-gel samples were reduced with DTT and then alkylated with iodoacetamide, washed, and then digested overnight with trypsin. After extraction, the tryptic digest was dried down and redissolved in 15 μL of 2.5% acetonitrile/0.1% formic acid. We ran 5 μL of the digest by nanoLC-MS/MS with a 2h gradient on a 0.075 mm × 250 mm Waters CSH C18 column feeding into a Q-Exactive HF mass spectrometer.

We analyzed all MS/MS samples with Mascot (Matrix Science, London, UKversion 2.6.1). Mascot was set up to search the cRAP_20150130.fasta (123 entries) and the Swiss-Prot database (selected for *Saccharomyces cerevisae* (7,905 entries) assuming trypsin digestion. Mascot was searched with a fragment ion mass tolerance of 0.060 Da and a parent ion tolerance of 10.0 PPM. Deamination of asparagine and glutamine, oxidation of methionine, and carbamidomethyl of cysteine, phosphoserine, threonine, and tyrosine were specified in Mascot as variable modifications.

Scaffold (version Scaffold_4.8.9, Proteome Software Inc.) was used to validate MS/MS-based peptide and protein identifications. Peptide identifications were accepted if they could be established at >80.0% probability by the Peptide Prophet algorithm (Keller *et al.* 2002) with Scaffold delta-mass correction. Protein identifications were accepted if they could be established at >99.0% probability and contained at least 2 identified peptides. Protein probabilities were assigned by the Protein Prophet algorithm (Nesvizhskii *et al.* 2003). Proteins that contained similar peptides and could not be differentiated through MS/MS analysis alone were grouped to satisfy the principles of parsimony. Proteins sharing significant peptide evidence were grouped into clusters.

### RNA isolation and cDNA synthesis

We used a RiboPure RNA purification kit (Invitrogen) to purify RNA from actively dividing cultures. The cells were lysed with a mini-bead beater (0.5 mm zirconia beads). Following isolation, the concentration of RNA was determined with a Nanodrop 2000 spectrophotometer. RNAs from WT and mutant strains were diluted to the same final concentration, and samples were frozen at -80°. Prior to performing cDNA synthesis and q-RT PCR, we treated the samples with 1µl of Invitrogen DNase I (amplification grade) as described in the protocol accompanying the kit. We carried out cDNA synthesis with an iscript cDNA synthesis kit (BioRad). We followed the BioRad protocol: 5 min of priming at 25° and reverse transcription for 20 min at 46°. Following this, the reverse transcriptase was inactivated for 1 min at 95° and the samples held at 4°.

### Protocol for q-RT PCR

We performed q-RT PCR with PowerUp SYBR green master mix (Thermofisher Scientific) according to the manufacturer protocol. The DNA polymerase was activated at 50° during a 2-min holding stage, followed by another 2-min holding stage at 95°. Amplification was achieved with 40 cycles at 95° for 15 sec and 60° for 1 min. The melting curve was performed for 15 sec at 95° and 1 min at 60°. The primers for *PDR5* and the three reference genes are found in the supporting information (Supplemental Material,Table S1).

### Metabolic labeling of mRNA with 4-thiouracil

For 4-thiouracil (4-TU) pulse experiments to evaluate transcription rate, logarithmic phase cells were grown to an O.D. 600 nm of 0.33 in SD medium containing 1 mM histidine and 0.1 mM uracil before adding 4-TU at a final concentration of 5mM. After 3 h growth at 30° in a shaking incubator, the cells were collected by centrifugation at 3000 × g at 4°. The cells were washed once and resuspended in a volume of SD containing 1 mM histidine and 20 mM uracil. The cultures were incubated at 30°. Aliquots of cells were removed at intervals of 0, 15, 30, and 40 min. The cells were washed once with 10 ml of cold phosphate-buffered saline, and after discarding the supernatant, the pellets were flash frozen in liquid nitrogen and stored at -80°.

To extract RNA, the cell pellets were thawed on ice for 20-30 min. RNA was extracted from up to 10^9^ cells/sample with a Ribopure yeast RNA extraction kit (Thermo Fisher). Following DNase treatment, RNA was quantified with a Nanodrop 2000 spectrophotometer and the concentration adjusted to 2 mg/ml. For each time point, 200-µg samples were stored for subsequent biotinylation and purification of 4-TU-labeled RNA at -80°.

### Thiol-specific biotinylation and subsequent recovery of 4-TU RNA

Biotinylation of 4-TU RNA was performed with an EZ-link HPDP-Biotin kit (Thermofisher Scientific) with minor modifications. A 200-μg aliquot of extracted RNA for each time point was heated for 1 min at 60° and chilled on ice for 2 min before transferring the material to a 2-ml spin tube. Following this, we added 600 μl of DEPC-treated, DNase- and RNase-free water and 100 μl of biotinylation buffer (final concentration was 100 mM Tris-HCl, 10 mM EDTA, pH 7.5, made up in DEPC-treated, DNase- and RNase-free water) and 200 μl of biotin-HPDP (1 mg/ml DMSO). The sample was incubated for 3 h at room temperature and protected from light. Following this, we added an equal volume of chloroform to the tube and mixed the contents vigorously. The sample was centrifuged at 13000 × g for 5 min at 4°, and the upper phase was transferred to 2-ml microcentrifuge tubes. We added 1/10 the volume of 5M sodium chloride and mixed the sample before adding an equal volume of isopropanol and centrifuging at 13000 × g for 35 min at 4°. The supernatant was removed and 1 ml of ice-cold 75% ethanol was added. The tubes were centrifuged at 13,000 × g for 10 min at 4°. The supernatant was removed and discarded. The tubes were recentrifuged for 30 sec to remove any residual alcohol. The pellets were not allowed to dry before they were resuspended in 100 μl of DEPC-treated, DNase- and RNase-free water.

The biotinylated RNA was heated for 10 min at 65° and the samples were chilled on ice for 5 min. One hundred µl of streptavidin-coated magnetic beads (Miltenyi Biotec) were added to the biotinylated RNA (volume 200 μl). The sample was incubated at room temperature with slight shaking for 90 min. The magnetic μ columns provided with the kit (Miltenyi Biotec) were placed in the magnetic MACS MultiStand, and 100 μl nucleic acid equilibration buffer was added. Following this, 900 μl of room-temperature biotynlation buffer (100 mM Tris-HCl [pH 7.5], 10 mM EDTA, 1 M NaCl in DEPC-treated, RNase-free water) was added to the columns. The bead/RNA mixture (200 μl) was then added to the columns. The flow-through was collected in 1.5-mL tubes and applied again to the same magnetic column. The flow-through was retained as it represented the unlabeled RNA fraction.

The columns were washed 5x with increasing volumes of washing buffer (600, 700, 800, 900, and 1,000 μl). The newly synthesized RNA was eluted with 200 μl of 0.1 M DTT. Three minutes later, a second elution was performed with an equal volume of 0.1 M DTT. After eluting the RNA, 0.1 volumes of 3 M NaOAc (pH 5.2), 3 volumes of ice-cold 100% ethanol, and 2 µl of 20 mg/mL glycogen, RNA grade (Thermo Scientific) were added, and the RNA was allowed to precipitate overnight, at -20°. The RNA was recovered by centrifugation (13,000 × g for 10 min, at 4°) and resuspended in 15 μl of DEPC-treated, RNase-free water.

### Statistical analyses

We analyzed the whole-cell and vesicle transport assays with Prism GraphPad software. Error bars in the figures or a ± designation in the text indicates the standard error of the mean.

### RNA folding structures

The predicted RNA secondary structures of WT and mutant linker-2 nucleotide sequences were obtained at unafold.rna.albany.edu using the Mfold web server for nucleic acid folding and hybridization prediction (Zuker 2003).

### Data availability

All of the strains and plasmids are available upon request. Table S1 contains a list of primers used in the q-RT PCR experiments. Figure S1 contains plots comparing WT and mutant resistances to imazalil sulfate and tamoxifen. Figure S2 contains plots comparing the resistance of the S837A mutant to the WT strain, JG2015. Figure S3 contains plots comparing S837A, S850A double mutant with their counterpart single mutant. Figure S4 contains plots comparing A666G mutant with S837A mutant growth in the presence of cycloheximide. Figure S5 compares the R6G transport capability of the S837A and S837D mutants to the WT strain. Figure S6 contains plots comparing the relative resistance to cycloheximide and climbazole of newly created WT (JG2015*) and S837A mutant strains. Figure S7 contains mRNA expression levels of Pdr5 from WT and S837A transformants. Figure S8 compares the RNA folding of linker-2 in WT and various mutant strains. Supplemental material available at figshare: https://doi.org/10.25387/g3.10287419.

## Results

### Alanine substitution mutants in six phosphoserine residues were hyper-resistant to Pdr5 substrates

**Figure 1:**
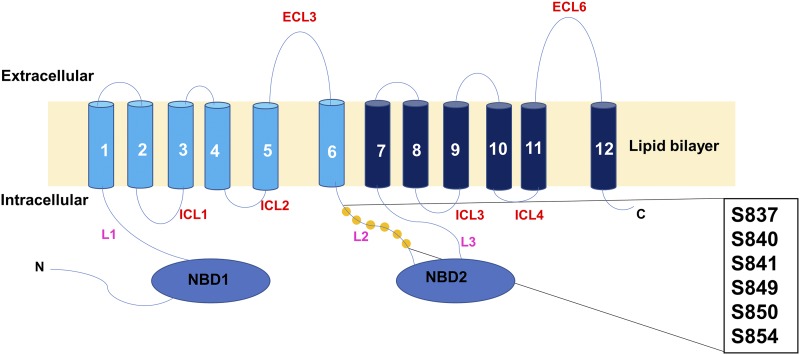
Relative location of the Linker-2 residues. The schematic illustrates the location of the six serine residues analyzed in this study. Linker (L)-2 connects TMH6 to the canonical portion of NBD2.

**Figure 2:**
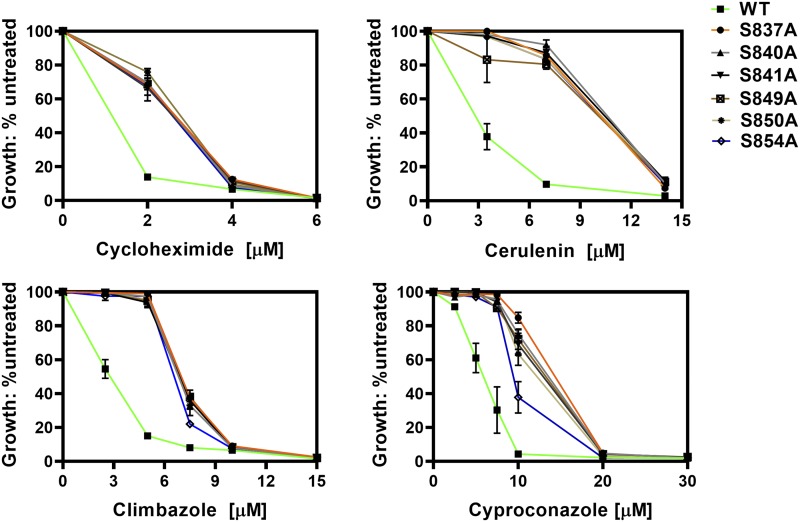
Preliminary testing of phosphoserine mutants indicated that they all increase drug resistance. Cultures of six alanine substitution mutants (JG2175-JG2181) were tested for resistance to clotrimazole, cycloheximide, cyproconazole, and cerulenin in liquid YPD cultures (48 h at 30 °C) at several concentrations as described in the Materials and Methods. In these experiments, n ≥ 4.

We constructed six single-alanine substitutions in the linker serines: S837A, S840A, S841A, S849A, S850A, and S854A. A schematic representation is found in Figure 1. The nucleotide changes that were made are found in Table 3. In this set of residues, four of six possible triplet codons for serine were used. At three of these positions (Ser-840, -850, and -854), a single nucleotide change resulted in the alanine substitution. It is therefore highly likely that such mutants arise *in vivo*. Our initial evaluation of drug resistance used climbazole, cycloheximide, cyproconazole, and cerulenin (Figure 2). Remarkably, all of the mutants were significantly more resistant than the WT control strain to all four compounds.

**Table 3:** Nucleotide changes resulting in alanine and aspartate substitutions in linker-2

Amino acid	Serine DNA codon	Substitution	New DNA codon
Ser-837	AGT	alanine	GCT
Ser-837	AGT	aspartate	GAT
Ser-840	TCC	alanine	GCC
Ser-841	AGC	alanine	GCC
Ser-849	AGC	alanine	GCC
Ser-850	TCT	alanine	GCT
Ser-854	TCC	alanine	GCC

**Figure 3:**
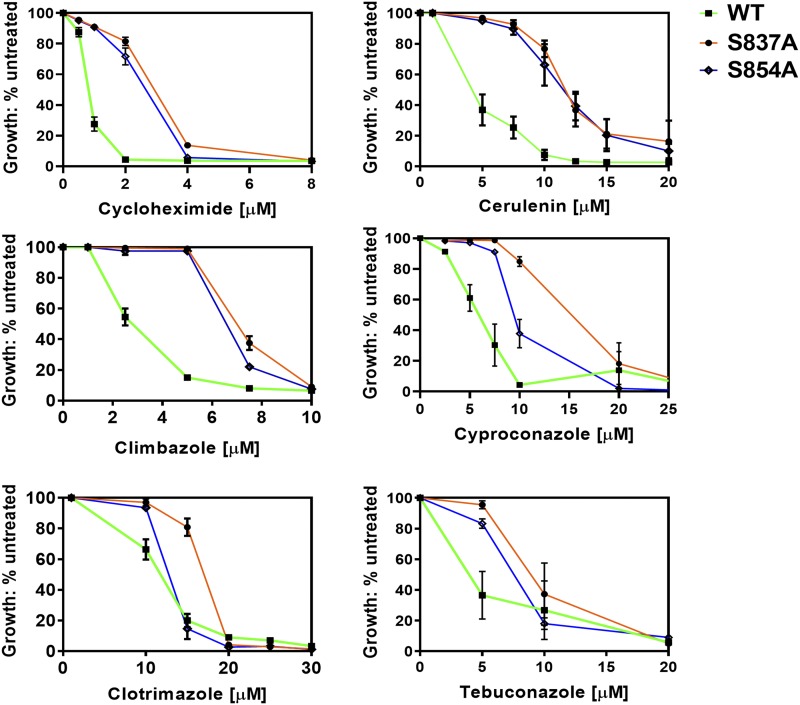
The S837A and S854A mutants exhibits strong hyper-resistance to multiple Pdr5 substrates. *Note*. ▪▪▪ = WT (JG2001, green line); ● = S837A (JG2175, orange line); ▪▪▪ = S854A (JG2181, blue line). Cells were cultured in YPD broth at 30 °C for 48 h in the presence of drugs, as described in the Materials and Methods. YPD cultures of each strain that contained no drug served as an untreated control for growth comparisons. Cell concentration was determined at 600 nm. (n ≥ 3).

We selected S837A and S854A for more in-depth study. We compared their relative resistance to eight Pdr5 transport substrates over a range of concentrations, and we used the isogenic WT strain as a control. The plots for six substrates are found in Figure 3; the remaining two are located in the Supplementary Information (Figure S1). The plots of the two mutants are similar and clearly demonstrate hyper-resistance relative to the WT strain. The S837A mutant exhibited modestly greater resistance to clotrimazole, tebuconazole and imazalil sulfate than did S854A. In general, the S837A mutant had estimated IC_50_ values that were about 2-4x as great as in the WT.

We noticed toward the end of this analysis that the WT strain (JG2001) that we used in this study was more sensitive to some of the transport substrates than the other WT strain that was also employed in our laboratory (JG2015, see Table 1 for strain history). When we sequenced the Pdr5 gene in the former, we observed no changes in the Pdr5 sequence. Nevertheless, we also compared the JG2015 and S837A strains for their relative resistance to six of the transport substrates (Figure S2). In each case, The S837A strain was significantly more resistant than the WT control. The JG2015 strain and its Ura- derivative (JG2015*; see text, Table 1, and Figure S2) were used exclusively in the whole-cell transport and biochemical experiments described below.

### An S837A, S854A double mutant does not exhibit greater resistance than the S837A and S854A single mutants

**Figure 4:**
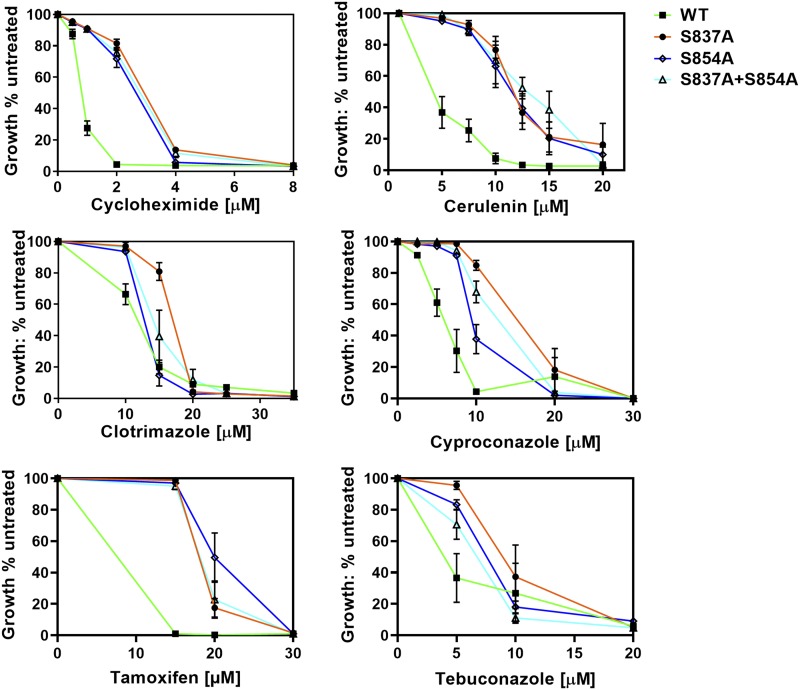
An S837A, S854A double mutant is no more hyper-resistant than single mutants. Resistance to xenobiotic agents was also monitored in single and double mutants as described in the Materials and Methods. Cells were cultured in YPD broth for 48 h at 30 °C. In these experiments, n ≥ 3. In all panels, the S837A (JG2175, orange line), S854A (JG2181, blue line), and double mutant (JG2182, brick red) were compared to each other and to the WT control (green line). We also tested the S850A mutant in combination with S837A with some of the drugs.

We initially constructed an S837A, S854A double mutant. We compared the resistance of single and double mutants to six of the substrates (Figure 4). These plots clearly demonstrate that in every case, the double-mutant strains had relative resistance that was no greater than either single-mutant counterpart. An analogous series of experiments with an S837A, S850A double mutant gave similar results for the four substrates that were tested (Figure S3).

One possibility we had to consider was that the single mutants reached the maximum resistance that Pdr5 could mediate for a substrate. If that were true, even mutations in two distinct biochemical steps would yield a nonadditive phenotype. This is clearly not the case. We tested the cycloheximide resistance of an A666G gain-of-function mutation that was previously characterized (Arya *et al.* 2019) along with the S837A mutant. The resistance of the former was roughly twice that of the latter (Figure S4). This demonstrated that Pdr5 is clearly capable of mediating a level of resistance that is greater than is exhibited by the S837A mutant.

### The S837A and S854A mutants exhibited enhanced R6G transport

**Figure 5:**
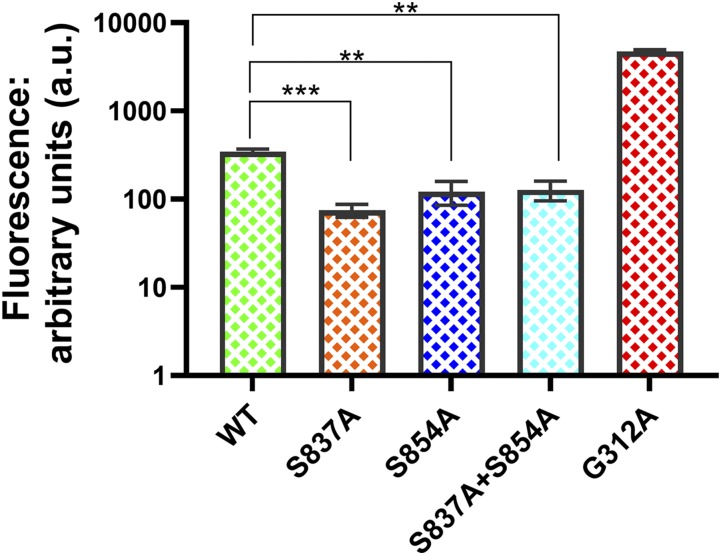
Rhodamine 6G transport is enhanced in the S837A and S854A mutant strains. R6G transport in whole cells was measured as described in the Materials and Methods. Incubations were performed for 90 min at 30 °C with 3 × 10^6^ cells in Hepes (0.0M) buffer containing 1 mM glucose and 10 μM R6G.The retained fluorescence in 10,000 cells was determined with a fluorescence cell sorter. In these experiments, n = 3.

We evaluated the R6G transport capability in whole cells of the S837A and S854A mutants relative to a WT and a G312A null mutant strain (Figure 5). The WT (JG2015) had a median fluorescence of 336.8 a.u., which was about 13.5x less than the catalytically dead G312A mutant (median value: 4539 a.u.). The median retained fluorescence values observed with the S837A and S854A mutants were 65.38 and 108.1, respectively. Thus, relative to the WT, these two mutants increased transport capability 5.2x and 3.1x, respectively. This differential was greater than most of those observed with other transport substrates when drug resistance was evaluated. An S837A, S854A double mutant showed the same level of retained fluorescence as the S854A mutant. A *t*-test found no significant difference in the fluorescence levels in the mutants.

### An S837D mutant strain was also hyper-resistant to Pdr5 substrates

**Figure 6:**
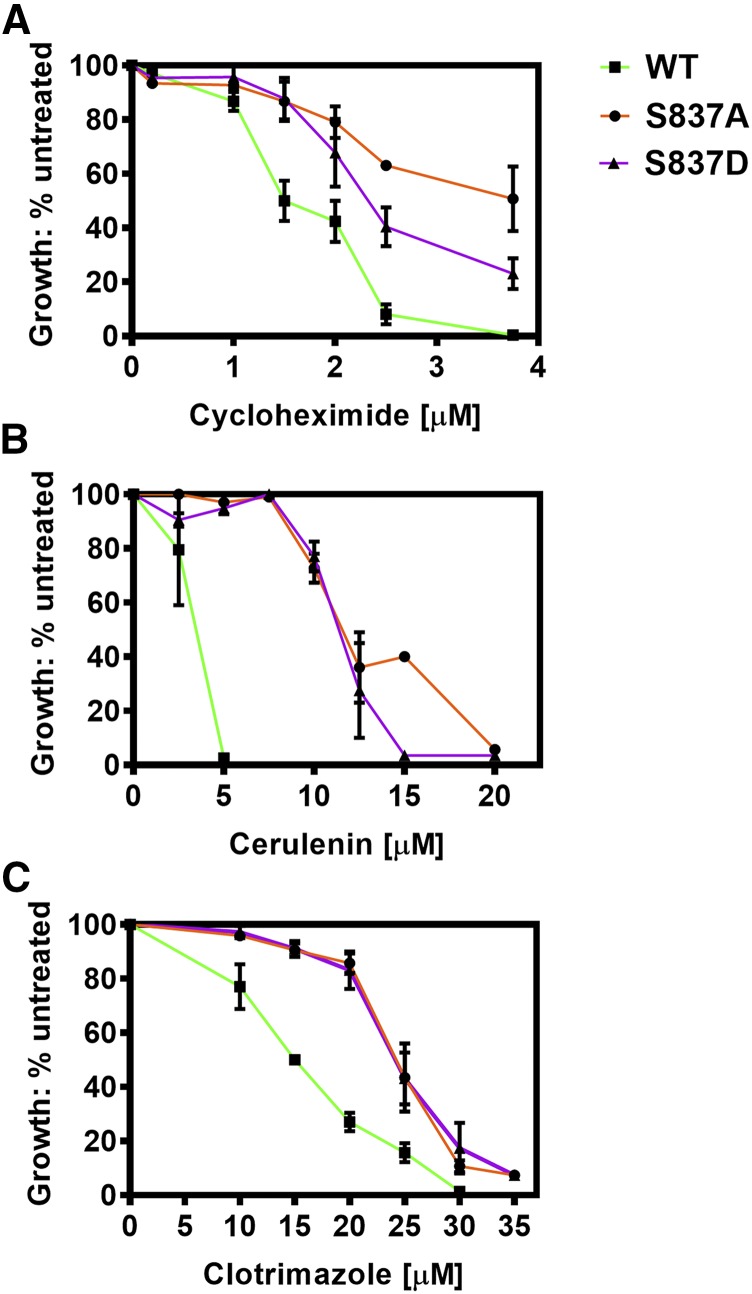
An S837D substitution mutant is also hyper-resistant to Pdr5 transport substrates. The S837D (JG2176, □, blue line) mutant strain was compared to the S837A mutant (JG2175, ●, orange line) and an isogenic WT control strain (JG2001, ▪▪▪, green line) for relative resistance to *(A)* cycloheximide and *(B)* cerulenin.

The use of serine-to-aspartate substitutions to create phosphomimics is a standard strategy. We made and tested an S837D substitution and predicted it would probably be phenotypically WT because Ser-837 is known to be phosphorylated from several mass spectrometry studies and because the S837A mutant, which can’t be phosphorylated, is hyper-resistant. However, our evaluation of the S837D mutant strain for resistance to clotrimazole, cycloheximide, and cerulenin revealed a phenotype similar to that of the S837A mutant (Figure 6).

We also compared the R6G transport capability of the S837D mutant to the S837A and WT strains (Figure S5). The S837D mutant retained significantly less fluorescence than the WT strain (*P* = 0.027).

These results were unexpected. We considered the possibility that the pSS607 plasmid where all of the mutants were constructed had acquired a mutation that increased resistance and was shared by all of the mutants. We therefore recovered the integrated S837D mutant sequence through PCR and sequenced the entire *PDR5* gene. We found no additional mutations.

### Mass spectrometry of Pdr5 phosphoserine residues suggested that Ser-837 is infrequently phosphorylated

We carried out mass spectrometry on Pdr5 peptide fragments to better evaluate the role of linker-2 phosphorylation on Pdr5 activity. Our observation that an alanine mutation at any of the six serines increased resistance indicated that the putative modulation by phosphorylation would require that most Pdr5 molecules be completely modified at these residues. We analyzed Pdr5 protein from purified PM vesicles prepared in the presence of phosphatase inhibitors.

First, we determined which serine residues were phosphorylated (Table 4). The mass spectrometry covered nearly 70% of the amino acids in Pdr5 (1040/1511). The majority of the phosphoserines were in the large amino-terminal tail and linker-2. The NetPhosYeast program (Technical University of Denmark) predicted that 14/20 had a greater than 50% probability of being phosphorylated. Four global phosphorylation studies of yeast phosphopeptides identified only 10/20.

**Table 4:** Twenty serine residues are phosphorylated in Pdr5

Number of phosphorylated serine	Location	Predicted probability	Present in global analyses (√)
17	Amino terminus	0.638	
18	Amino terminus	0.600	
20	Amino terminus	0.519	
21	Amino terminus	0.674	
22	Amino terminus	0.595	**√**
54	Amino terminus	0.791	**√**
58	Amino terminus	0.639	**√**
61	Amino terminus	0.673	**√**
70	Amino terminus	0.501	
74	Amino terminus	0.290	
104	Amino terminus	0.203	
126	Amino terminus	0.073	
318	NBD-1	0.573	
**837**	**Linker 2**	**0.542**	**√**
**840**	**Linker 2**	**0.514**	**√**
**841**	**Linker 2**	**0.444**	**√**
**849**	**Linker 2**	**0.667**	**√**
**850**	**Linker 2**	**0.755**	**√**
**854**	**Linker 2**	**0.427**	**√**
945	NBD2	0.224	

Further analysis of the results from mass spectrometry revealed 10 distinct phosphopeptides that included Ser-849, 850, and 854 and had a high probability (> 99%) of matching Pdr5 in the database (there is a trypsin site between Ser-841 and Ser-849). The relative abundance of a particular pattern was estimated from the area of each peptide’s chromatographic peak. Ser-850 was phosphorylated in all cases; Ser-854 in a majority of the cases. For the sequence covering Ser-837, Ser-840, and Ser-841, three distinct cases were recovered. None of the three phosphopeptides was phosphorylated at all three sites. The two most abundant phosphopeptides lacked a phosphoserine at residue 837. Although other explanations are plausible, these data suggested that a significant portion of the Pdr5 molecules present *in vivo* contain at least one linker-2 serine that remains unmodified.

### Increased levels of Pdr5 in purified PM vesicles in the S837A mutant strain

**Figure 7:**
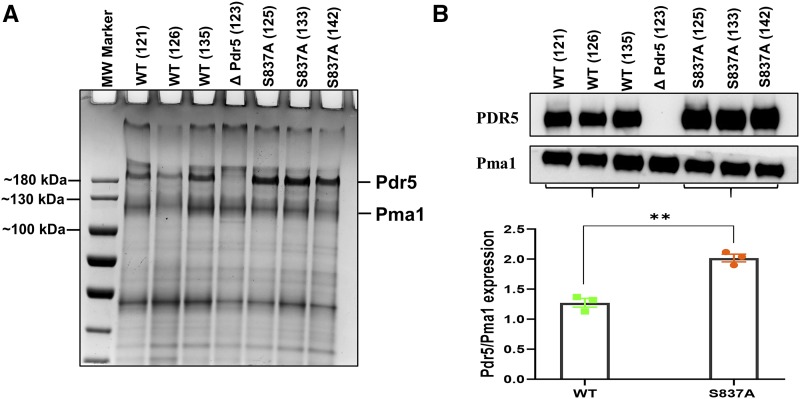
Plasma membrane vesicles made from the S837A strain have more Pdr5 in the membrane. Purified PM vesicles were prepared as described by Kolaczkowski *et al.* (1996) and modified by Ernst *et al.* (2008). *(A)* PM (6 μg) protein samples from three preparations of S837A (JG2175) and three preparations of WT (JG2015) PM vesicles were solubilized in SDS PAGE and subjected to electrophoresis in 7% tris-acetate gels for 80 min at 150 V before staining with coomaissie blue. *(B)* Samples (3 μg) from the same PM vesicle preparations were also immunoblotted as described in the Materials and Methods.

Over the course of these studies, we made three independent PM vesicle preparations from the S837A mutant and from the WT strain. Gel electrophoresis was performed, and the separated proteins were stained with Coomassie blue. The uniform density of the Pma1 band (except in the ΔPdr5 lane) across the gel indicates equivalent loading of solubilized protein from each sample. The S837A PM vesicles clearly contained more overexpressed Pdr5 than the controls (Figure 7A). This result was also confirmed by Western blotting of PM vesicles (Figure 7B). When the Pma1 proton pump was used as a loading control, the median Pdr5/Pma1 ratio in the WT was 1.32 (1.13 - 1.17). The median ratio observed in the mutant preparations was 2.04 (1.90 - 2.12) or 1.55x the WT value. The difference was highly significant when an unpaired *t*-test was performed (*P* = 0.0016).

**Figure 8:**
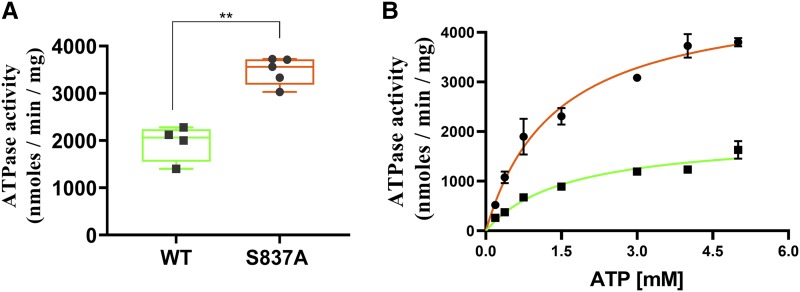
The ATPase activity is doubled in PM vesicles prepared from the S837A mutant. PM vesicles were prepared as described by Kolaczkowski *et al.* (1996). *(A)* The assay of ATPase activity was performed as previously described (Arya *et al.* 2019) with 3 mM ATP. Activity was measured in Tris-glycine buffer (pH 9.5). Activity was assayed in two different PM vesicle preparations prepared from the S837A mutant (JG2175) and three from the WT strain, JG2015. *(B)* ATPase activity was measured as a function of ATP concentration in PM vesicles prepared from WT (▪▪▪, green line) and S837A (●, orange line) strains in the conditions described in the Materials and Methods.

These results also suggested that the PM vesicles prepared from the S837A mutant strain would have about twice the ATPase activity of the WT control. This was the case when we assayed independent preparations for ATPase activity with 3mM ATP (Figure 8A). The median activity of mutant PM vesicles at this concentration was ∼1.8x as high as the WT (3.6 and 2.0 μmol/min/mg, respectively). We also measured ATPase activity with varying ATP concentrations with a WT and S837A mutant preparation. As expected, the kinetics fit the Michaelis-Menten equation. The V_max_ for the WT and S837A mutant preparations were 1.9 and 4.8 μmol/min/mg respectively (Figure 8B). The increase in ATPase activity was therefore proportional to the increased level of Pdr5.

**Figure 9:**
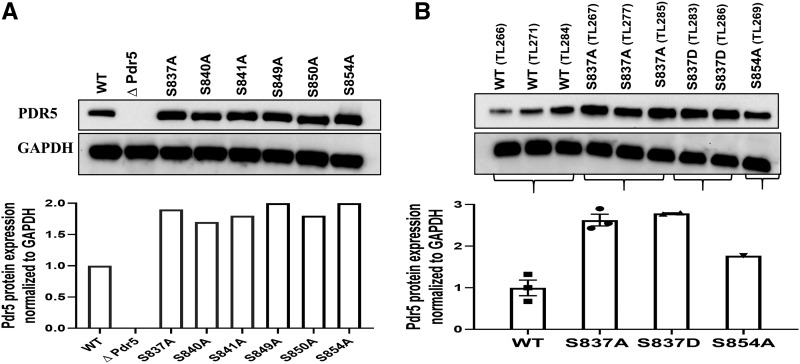
The alanine mutants exhibited elevated levels of Pdr5 in whole-cell extracts. *(A)* Whole-cell extracts were prepared, proteins (10 μg/sample) were solubilized in SDS PAGE, and gel electrophoresis and Western blotting were performed as described by Rahman *et al.* (2018). The relative amounts of Pdr5 were determined and normalized with GAPDH as the loading standard. The numbers at the bottom of each lane represent the ratio of mutant to WT (JG2015) Pdr5 protein. *(B)* Extracts were also prepared from the S837D mutant (JG2176) and, for comparison, from the WT, S837A (JG2175), and S854A (JG2181) strains. They were blotted as described in the Materials and Methods and in panel A. The bar graph contains the average enhancement found in multiple WT, S837A, and S837D blots.

We also monitored the level of Pdr5 in whole-cell extracts prepared from the WT and all of the alanine substitution mutants (Figure 9A). If the increased resistance was caused by improved trafficking to the PM, the enhancement in Pdr5 level would not have been the same in a whole-cell lysate, where PM vesicles are not the only source of Pdr5 protein. In all cases, the enhancement for all of the mutants was about twice the WT level. In a separate experiment (Figure 9B), we also blotted samples from two independent preparations of the S837D mutant, along with a lysate from S854A and several from S837A and the WT. In this set of experiments, the S837D and S837A mutants showed a comparable level of enhancement. In contrast, the level of Pdr5 in the S854A extract was lower than those observed with the other mutants, but clearly higher than the WT.

### The S837A mutant does not increase the stability of Pdr5

An obvious potential explanation for the increased expression of alanine and aspartate substitution mutants was that they had increased the stability of Pdr5. Two relatively early studies of Pdr5 turnover that used pulse-chase experiments yielded different estimates of its half-life. It is possible that the different genetic backgrounds used in the two studies were responsible. The first (Egner and Kuchler 1995) indicated the Pdr5 half-life was 60-90 min; the second (Decottignies *et al.* 1999) showed that it was in excess of 2 h.

**Figure 10:**
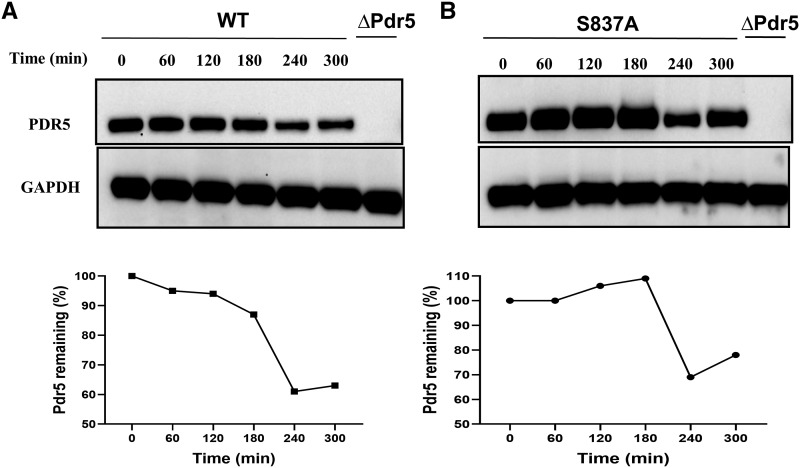
Pdr5 is a relatively stable protein. A cycloheximide chase experiment was performed with the WT (JG2015) *(A)* and S837A (JG2175) *(B)* mutant cells as described in the Materials and Methods. Following the addition of 125 μM cycloheximide to the cultures, aliquots of cells were lysed at 1-h intervals and subjected to electrophoresis and Western blotting. *(C)* The amount of Pdr5 protein was normalized with respect to GAPDH protein that was run on the same gel. The filter was cut and the Pdr5 and GAPDH signals developed separately. A second time course experiment reproduced the results in this figure.

We performed a cycloheximide-chase experiment to determine whether increased stability of the S837A mutant was a reasonable explanation for the increased level of mutant Pdr5 in the PM (Figure 10A, B). The cell-division time for the WT strain was ∼90 min. There was no reduction in the level of Pdr5 for at least 2 h. A significant decrease in the amount of Pdr5 was not observed until the 4-h time point. At 5 h, roughly 63% and 72% of the WT and S837A protein respectively, remained. A repeat of the experiment gave similar results, although there was no difference between the WT and S837A mutant preparations. It didn’t seem likely that increased stability of mutant proteins was responsible for the hyper-resistant phenotype of the alanine substitutions.

### The S837A mutant has a higher level of Pdr5 mRNA

**Figure 11:**
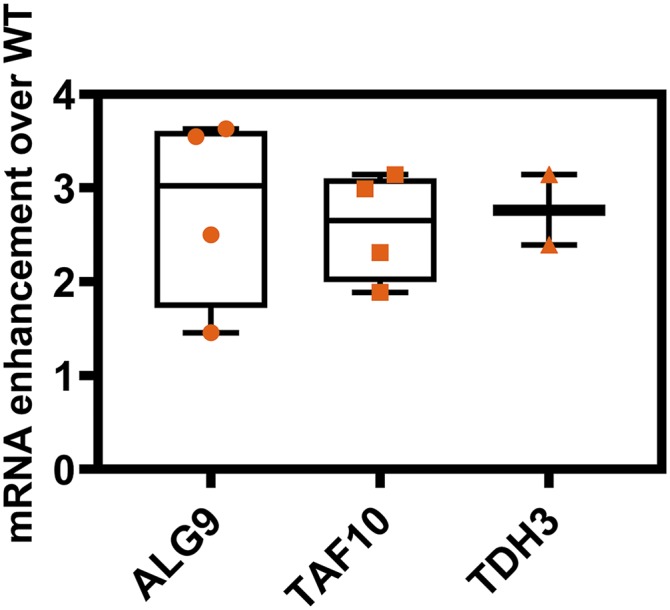
The S837A mutant has enhanced levels of *PDR5* transcript. Q-RT-PCR was performed as described in the Materials and Methods. The ΔC_T_ values for the three references genes were determined and these values were used to obtain the ΔΔC_T_ between the WT and mutant strains for each reference gene. The times difference was calculated from the formula: fold change = 2^ΔΔC^_T_. For the *ALG9* and *TAF10* experiments, n =4. For the *TDH3* determination, n =3.

The cycloheximide chase experiment strongly indicated that the increased levels of Pdr5 could not be attributed to enhanced stability of the transporter. We therefore considered the possibility that these mutants had enhanced levels of Pdr5 transcript. We used q-RT PCR to compare the levels in the WT and mutant with three reference genes: *ALG9*, *TAF10*, and *TDH3* (Teste *et al.* 2009). Following this, we calculated the difference between the strains from the ΔΔCT for each reference gene and averaged the three values before calculating the enhancement with the 2^-ΔΔc^_T_ method (Livak and Schmittgen 2001). The high C_T_ for the negative controls, which lacked reverse transcriptase, indicated that we were amplifying mRNA rather than contaminating DNA (which was eliminated by DNAse treatment). The results from four independent experiments (Figure 11) indicated that the S837A mutant showed an enhancement that was similar in magnitude to the enhancements in Pdr5 level, drug resistance, and R6G transport that we observed in the alanine substitution mutants. When the *ALG9* gene was used as the reference, the 95% confidence limits indicated an enhancement range of 2.50 to 3.63 times the WT (median = 3.37x). The 95% confidence limits for *TAF10* gave a range of 1.89 to 3.14 times the WT (median = 2.65x); those values for *TDH3* were 2.39 to 3.15 times the WT (median = 3.02x).

### The S837A mutation increases the half-life of the *PDR5* transcript

**Figure 12:**
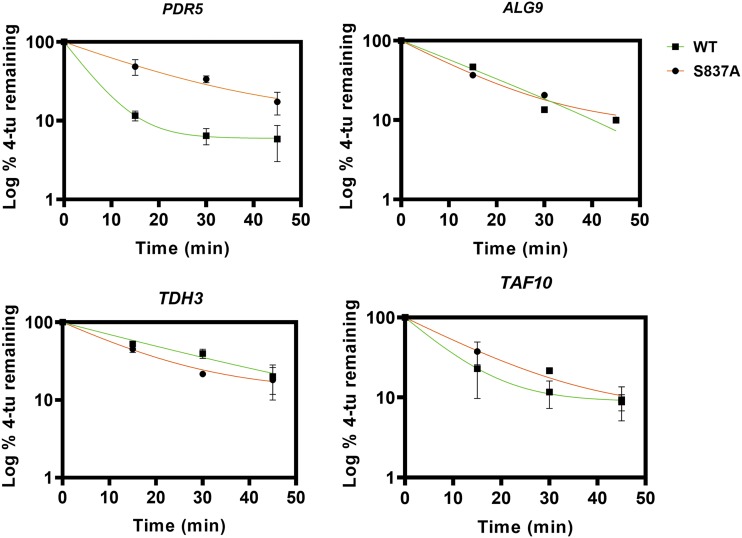
The S837A mutation increases the *PDR5* transcript half-life. RNA was labeled continuously with 4-TU for three hrs. Following this, cells were chased with 5 mM uracil for various times before extracting the RNA and recovering the 4-TU-containing transcripts as described in the Materials and Methods. Following q-RT PCR, the C_T_ values were used to calculate the % 4-TU remaining at 15, 30, and 45 min with the value at 0 min as 100%. Data were plotted using the one-phase exponential decay equation: y= span e^-k.x^ + plateau where k is the rate constant, x is the time, y is the log % 4-tu remaining after the uracil chase. In all of these plots, ▪▪▪ = WT (JG2015, green line); ● = S837A (JG2175, orange line). In these experiments, n = 3.

A possible explanation for the increased *PDR5* transcript levels was that their half-lives were extended by these mutations. To evaluate whether this was the case with the S837A mutant strain, we labeled RNA continuously with 4-TU and chased with cold uracil for up to 45 min with samples taken at 15-min intervals. RNA molecules containing 4-TU were recovered by crosslinking and column purification as described in the Materials and Methods. We then performed q-RT PCR and determined the C_T_ for *PDR5* and the three reference genes. The amount of transcript remaining relative to the time zero point (100%) was determined from three independent experiments and plotted with a one-phase exponential decay equation (Figure 12). The R^2^ values exceeded 90% in all cases (and 99% in a majority). Comparison of the plots of the mutant and WT versions of the three reference genes revealed that the small differences in their half-lives were not significant. In contrast, however, the half-life of the *PDR5* transcript was 3.4 times as long as in the mutant (12.7 min) as in the WT (3.70 min). The 95% confidence intervals did not overlap. A two-way ANOVA test indicated that the row factor (comparison between strains of the remaining 4-tu at each time point) was a source of significant variation (*P* = 0.0045) This suggests that the increase in steady-state level can be completely attributed to the increased half-life of the S837A transcript.

### The hyper-resistance exhibited by the mutants is not attributable to a non-*pdr5* mutation in the R-1 strain or pSS607 plasmid

The JG2015 WT strain was constructed from R-1 (Table 2) and has been used in our laboratory since 2007. The S837A strain was constructed in R-1 in 2017. One explanation for the similar phenotypes of the seven mutants analyzed in this study is that their hyper-resistance was caused by another alteration that took place during the 10-year interval separating these strains. This seemed unlikely because non-linker 2 mutants made in pSS607 and placed in R-1 after S837A was constructed had WT levels of Pdr5 in PM vesicles. Nevertheless, to be sure, we retransformed the strain with the pSS607 and pS837A plasmids. We compared the two original strains and two WT (JG2015*) and S837A mutant transformants for resistance to cycloheximide and climbazole (Figure S6), These had phenotypes that were indistinguishable from their original counterparts.

The expression level of the *PDR5* transcript was evaluated in a WT and a new S837A transformant. The latter exhibited enhanced expression when the *ALG9* and *TAF10* genes were used as references (Figure S7).

## Discussion

When we initiated this study, we assumed that the hyper-resistant alanine mutants were the result of a lack of phosphorylation at the missing serine residue. Remarkably, all six of these mutants had increased levels of Pdr5. A possible explanation was that linker-2 modulated Pdr5 activity by its reduction through phosphorylation of these serines. Several observations ruled out this explanation. Modulation would have required that many copies of the Pdr5 transporter be phosphorylated at all six residues, but the mass spectrometry data indicated that some residues—notably Ser-837–were often missing a phosphate group. Furthermore, the S837A and S837D mutants were both hyper-resistant to several Pdr5 substrates.

Instead, we observed that the increase in steady-state levels of Pdr5 in purified PM vesicles could be explained by the surprising observation that the S837A mutant had a higher steady-state level of mRNA. Experiments with the uracil analog 4-TU demonstrated that the enhancement observed in the mutant was attributable to an increased half-life of the *PDR5* transcript. Our results are in reasonably good agreement with a global analysis of WT yeast half-lives that also used the 4-TU technique (Chan *et al.* 2018). Those investigators calculated a *PDR5* half-life of 4.35 min, which is similar to our observed value (3.70 min). Their values for *TAF10* (6.11 min) and *TDH3* (23.4 min) were also reasonably close to the values we obtained for these transcripts (5.58 and 18.5 min, respectively). Our estimated half-life for *ALG9*, however was significantly longer than theirs (12.8 *vs.* 3.67 min). The reason for this disparity is not obvious.

Yeast mRNA half-lives are the subject of numerous studies in many genes. Global studies of yeast transcription identified crucial sequences in both the 3′ untranslated portions and the 5′ end (Shalgi *et al.* 2005; Geisberg *et al.* 2014). Translation initiation is also extremely important (Chan *et al.* 2018). As noted in the introduction, it is also well established that some synonymous mutations affect transcription rate, splicing, or stability.

Reports of missense mutations affecting transcription, however, are not found in abundance. Two studies with other ABC transporters are of interest. Manoharial *et al.* (2008) demonstrated that Cdr1 mutants selected for increased resistance to azoles exhibited both increased transcription rate and mRNA stability. These investigators ruled out alterations in the promoter regions but did not provide sequencing data. Thus, it was not clear whether these phenotypes were attributable to synonymous or nonsynonymous alterations or whether more than one mutation was involved. However, a study using *Pseudomonas fluorescens* was particularly revealing (Bailey *et al.* 2014). When a strain of this bacterium was grown for 1000 generations with growth-limiting concentrations of glucose both spontaneous synonymous and nonsynonymous mutations were recovered in the ABC glucose transporter permease subunit that increased transcription. Although the mechanism behind the increase, was not determined, these data clearly show that positive selection acts at the nucleotide level. There is also a report of a missense mutation in the mouse inositol-5-phosphatase gene that reduces transcription and creates a fairly profound loss-of-function phenotype (Nguyen *et al.*2011). It may be the case that most bifunctional codons are found in relatively unconserved amino acids where mutations are less likely to cause significant perturbations in protein structure. These changes would be similar to synonymous mutations. Because unconserved residues are not studied as extensively as conserved ones, missense mutations affecting transcription in the former might be overlooked.

Linker-2, a region of approximately 60 amino acids connecting TMD-1 (via TMH6) to the canonical portion of NBD2, is not well conserved. For instance, Pdr5 shares 75% and 67% amino acid identity respectively with its two paralogs Pdr10 and Pdr15. Nevertheless, the amino acid identity in the linker-2 region is only 47% and 45% respectively in these transporters. In both cases, amino acid substitutions are found in the positions corresponding to the Pdr5 serines that we analyzed. In Pdr10, the residues equivalent to Ser-840 and Ser-841 are replaced with an alanine and glutamic acid. The changes in Pdr15 are even more dramatic. Ser-837 is replaced by proline, and the end of linker-2 containing Ser-449 to Ser-854 is missing entirely. Among the 12 strains the *Saccharomyces* Genome Database surveyed for variants, four alterations were found in the linker-2 region among three strains (although none were in the six serines). This information, and the surprising observation that seven substitutions in six serine residues create similar resistance phenotypes gives rise to some interesting speculation. First, these observations suggest that unconserved nucleotides may be fertile ground for regulating mRNA abundance through altered secondary structure.

Furthermore, it would appear that the shorter WT *PDR5* transcript half-life is the default, favorable option. This assumes that the mechanism for the increased resistance of the Ala-837 mutant applies to the other substitutions as well. Their *PDR5* half-lives were not determined in this study. The observation that double mutants are no more resistant than single mutants is also consistent with the idea that the short half-life of *PDR5* transcript is the result of a particular, naturally selected secondary structure. Many departures from that structure in the linker-2 region of the RNA molecule presumably default to longer half-life regardless of whether there are one (S841A or S854A, for example), two (S837A), or three (S37A, S854A) altered nucleotides. In the Supplementary Information, we provide secondary structures of linker 2 RNA made from the RNA Institute software (Figure S8). Our data suggest that some of the linker-2 codons define an RNA stability element that reduces the lifetime of the *PDR5* transcript.

There are at least two, non-mutually exclusive plausible explanations for the short half-life of the *PDR5* transcript. The first is prompted by the kinetics of Pdr5 ATPase activity. A striking feature of the activity is that although the K_m_ (ATP) is quite high (about 2 mM), the basal ATPase activity is enormous (roughly 2 µmole P_i_ released / mg / min) in strains that overexpress this protein. This means that the transporter is using a substantial quantity of ATP. This might be detrimental especially under nutrient-limiting conditions where Pdr5 is known to function in robust fashion (Rahman *et al.* 2018). Perhaps continued overexpression of the transporter is selectively disadvantageous over time even though it results in increased resistance. Alternatively, overexpressed Pdr5 might deplete the cell of important metabolites. These ideas are consistent with the observation that *PDR5* transcription is temporarily elevated in the presence of a drug via the Pdr1 transcriptional regulator (Fardeau, *et al.* 2007). Thus, in most common yeast strains, Pdr5 production appears to be highly regulated.
